# Effect of the 6-week home-based exercise program on physical activity level and physical fitness in colorectal cancer survivors: A randomized controlled pilot study

**DOI:** 10.1371/journal.pone.0196220

**Published:** 2018-04-26

**Authors:** Mi Kyung Lee, Nam Kyu Kim, Justin Y. Jeon

**Affiliations:** 1 Department of Sport Industry Studies, Exercise Medicine and Rehabilitation Laboratory, Yonsei University, Seoul, Republic of Korea; 2 Exercise Medicine Center for Diabetes and Cancer Patients, ICONS, Yonsei University, Seoul, Republic of Korea; 3 Department of Surgery, Yonsei University College of Medicine, Seoul, Republic of Korea; 4 Cancer Prevention Center, Yonsei University College of Medicine, Seoul, Republic of Korea; University of Rochester, UNITED STATES

## Abstract

Despite improvement in prognosis of colorectal cancer, colorectal cancer survivors often suffer from adverse effects of cancer treatment, including reduced health-related fitness level. Therefore, this study aimed to examine the feasibility and efficacy of the 6-week home-based exercise program on the level of physical activity and physical fitness in stage II to III colorectal cancer survivors. Seventy-two stage II to III colorectal cancer survivors were randomly assigned to either a home-based exercise (n = 38) or usual care (n = 34) group for 6 weeks. The goal of the home-based exercise program was to increase the level of exercise to 18 metabolic equivalent task hours per week. The primary and the secondary outcomes of this study were physical activity level and physical fitness, respectively. A total of 57 participants (79.2%) completed the trial. Intention-to-treat analysis indicated that moderate physical activity level increased significantly by 269.4 ± 260.6 minutes per week in the exercise group (mean between-group difference, 254.6 minutes; 95% confidence interval, 172.7–434.7; *p* < 0.001). Physical fitness measured by using the step test (-3.9 vs. 2.6, *p* = 0.012) and push-up test (3.0 vs. -1.2, *p* = 0.012) also improved significantly in the exercise group compared to the control group. The 6-week home-based mixed aerobic and resistance exercise program was feasible and effective for increasing physical activity level and physical fitness in stage II to III colorectal cancer survivors.

## Introduction

Colorectal cancer survival rates are continuously improving due to early detection and improved health care. In Korea, the 5-year relative survival rate of patients diagnosed with colorectal cancer from 2008 to 2012 was 74.8% (76.9% in men, 71.8% in women) [1]. Despite improvement in survival, colorectal cancer survivors often suffer from the adverse effects of cancer treatment (surgery and/or chemotherapy), including reduced health-related fitness level, [2] functional capacity [1, 3], and quality of life, [4] as well as increased fatigue [5–7].

Physical activity is not only associated with reduced risk of recurrence and cancer-related death from colorectal cancer [8–10], but it also improves cardiorespiratory fitness, strength, fatigue, depression, and quality of life in cancer survival [11–14]. Despite the benefits of physical activity, it has been reported that only 23.5% of colorectal cancer survivors participate in over 150 minutes of moderate to vigorous physical activity per week [15]. Barriers of participation in exercise among colorectal cancer survivors include fatigue, low level of physical fitness, and poor health [16].

Recently, a meta-analysis including 87 patients from 3 randomized controlled trials provided strong evidence for short-term improvement in physical fitness after exercise interventions in colorectal cancer survivors compared with controls [11]. However, further evidence regarding the efficacy and safety of exercise interventions for colorectal cancer survivors is required. Although most cancer patients agree that participation in exercise is important for their health, many patients experience difficulties in finding and affording a supervised exercise program tailored for cancer patients. One of the ideal solutions to increase physical activity and exercise participation is the development and providence of home-based exercise programs. There are limited information on feasibility and efficacy of a home-based mixed aerobic and resistance exercise program for colorectal cancer survivors. Therefore, the purpose of this study was to examine the feasibility and efficacy of the 6-week home-based exercise program with regard to physical activity level and physical fitness in stage II to III colorectal cancer survivors.

## Materials and methods

### Setting and participants

The participants were 72 colorectal cancer survivors who were recruited from Yonsei Severance Hospital, Seoul, Korea, February to December 2012. Eligibility criteria were as follows: age between 18 and 75 years, histologically confirmed stage II to III colorectal cancer with completed surgery, radiotherapy, and/or chemotherapy within 4 weeks to 2 years prior to study enrollment, and ability to understand and provide written informed consent in Korean. Exclusion criteria were as follows: existing evidence of recurrent or metastatic disease, history of ostomy, pregnant or planned to become pregnant within 6 months, and current participation in >200 minutes of moderate to vigorous activity per week.

### Design and procedure

This pilot study was a 2-armed, randomized and controlled trial comparing an exercise group and a usual care group. Interested participants were screened by telephone for eligibility. Participants were randomly assigned to the exercise or usual care group by a biostatistician using the Research Randomizer website program (https://www.randomizer.org) in a 1:1 ratio with block randomization based on gender. The study protocol was approved by the Institutional Review Board of Severance Hospital, and all participants provided the written informed consent.

### Intervention

The focus of the intervention was to increase participants’ level of exercise to more than 18 metabolic equivalent task (MET) hours per week over 6 weeks. This amount of exercise equates to approximately 6 hours per week at a moderate intensity walk. Participants in the exercise group were prescribed with a home-based exercise program with an exercise diary and pedometer. Brisk walking, hiking and stationary bike riding were recommended for aerobic exercise. In addition, patients were provided with videos that contained two 30 minutes resistance exercise programs using their own bodyweight of different intensities that could be performed daily at home. For mild intensity exercise, participants performed 3 sets of 15 repetitions of 7 core & resistance exercises and for moderate intensity exercise, participants performed 3 sets of 15 repetitions of 5 combined aerobic & resistance exercises. This home-based exercise program was developed based on the survey results of 431 colorectal cancer survivors [15, 16]. Participants in the exercise group had three clinic visits at baseline, at 1st week and at 6th week while participant in the control group had two clinic visits (at baseline and at 6th week). During the counseling session (at baseline and at 1st week), participants learned about benefits of exercise for colorectal cancer patients as well as how to perform exercise correctly and follow the home-based exercise program. In order to increase compliance, a text message was sent to each participant daily. The text message includes a question that participants were required to answer using 1 of the 3 following options: (1) Yes, I have completed my daily exercise, (2) No, I have not completed my daily exercise, but I will, or (3) No, I have not completed my daily exercise, and I am not be able to complete it today. Participants with response rate of 50% to the text message were followed up with telephone counseling.

Participants in the usual care group were instructed to continue with their usual activities. At the end of the study, the usual care group was provided with exercise videos, a pedometer, and exercise program.

### Measurements

Primary outcome measurement (Physical activity): Level of physical activity was assessed by using the validated Leisure Score Index from the Godin Leisure-Time Exercise Questionnaire [17]. Participants were asked to recall their average weekly frequency and duration of mild-, moderate-, and strenuous-intensity exercise that lasted at least 10 minutes and was done during their free time in the past week [18]. The totals for each intensity were calculated, along with total exercise time per week and MET hours per week (strenuous, 6 MET hours; moderate, 4 MET hours; mild, 3 MET hours).

Secondary outcome measurements (Physical fitness and body composition): Physical fitness was evaluated by using the Tecumseh step test, the 6-minute walk test, the 30-second chair-to-standing test, and the push-up test. During the step test, participants were asked to step up and down on a 20.3-cm-high bench for 3 minutes at a rate of 24 steps per minute, as controlled by a metronome. Immediately following the 3 minutes of exercise, participants rested for 1 minute in a sitting position. Participants’ heart rate was recorded after 1 minute of rest. For the 6-minute walk test, participants were asked to walk for a total of 6-minutes and to cover as much distance as possible. After 6 minutes, the total walking distance was recorded. For the 30-second chair-to-standing test, participants were asked to sit and rise with fully sitting and standing positions without using the arms for support as many times as possible in 30 seconds. For the push-up test, male participants were asked to maintain their body and legs in a straight line, their feet slightly apart, and their arms shoulder width apart, extended, and at a right angle to the body. Female participants performed bent-knee push-ups, which begin with the hands and knees touching the floor with the body and legs in a straight line and with the feet raised in the air. Keeping the back and knees straight, participants lowered their body until there was a 90° angle at the elbows and then returned back to the starting position with the arms extended. This motion was repeated until the participant could no longer perform push-ups in rhythm.

Body composition including percent body fat was measured by bio-impedance device, In-body 720 (Biospace, Seoul, Korea). BMI was calculated as weight divided by height squared. WC was measured at the midpoint between the lower border of the rib cage and the iliac crest using a plastic tape measure. All these measurements were measured at baseline and after six week of exercise intervention.

### Statistical analysis

Data were analyzed using SPSS version 22.0 (IBM Corporation, Armonk, NY). Baseline comparisons were performed by using the independent-samples Student’s *t* test for continuous variables and the chi-square test for categorical variables. Study endpoints were analyzed by using the ANCOVA to compare changes in means between groups from baseline and 6 weeks, adjusting for age, gender, body mass index, completed university, location of tumor, tumor stage, and cancer treatment according to intention-to-treat principles using the last-observation-carried-forward method. All analyses were tested with a significance level of *p* < 0.05.

## Results

**Fig 1:**
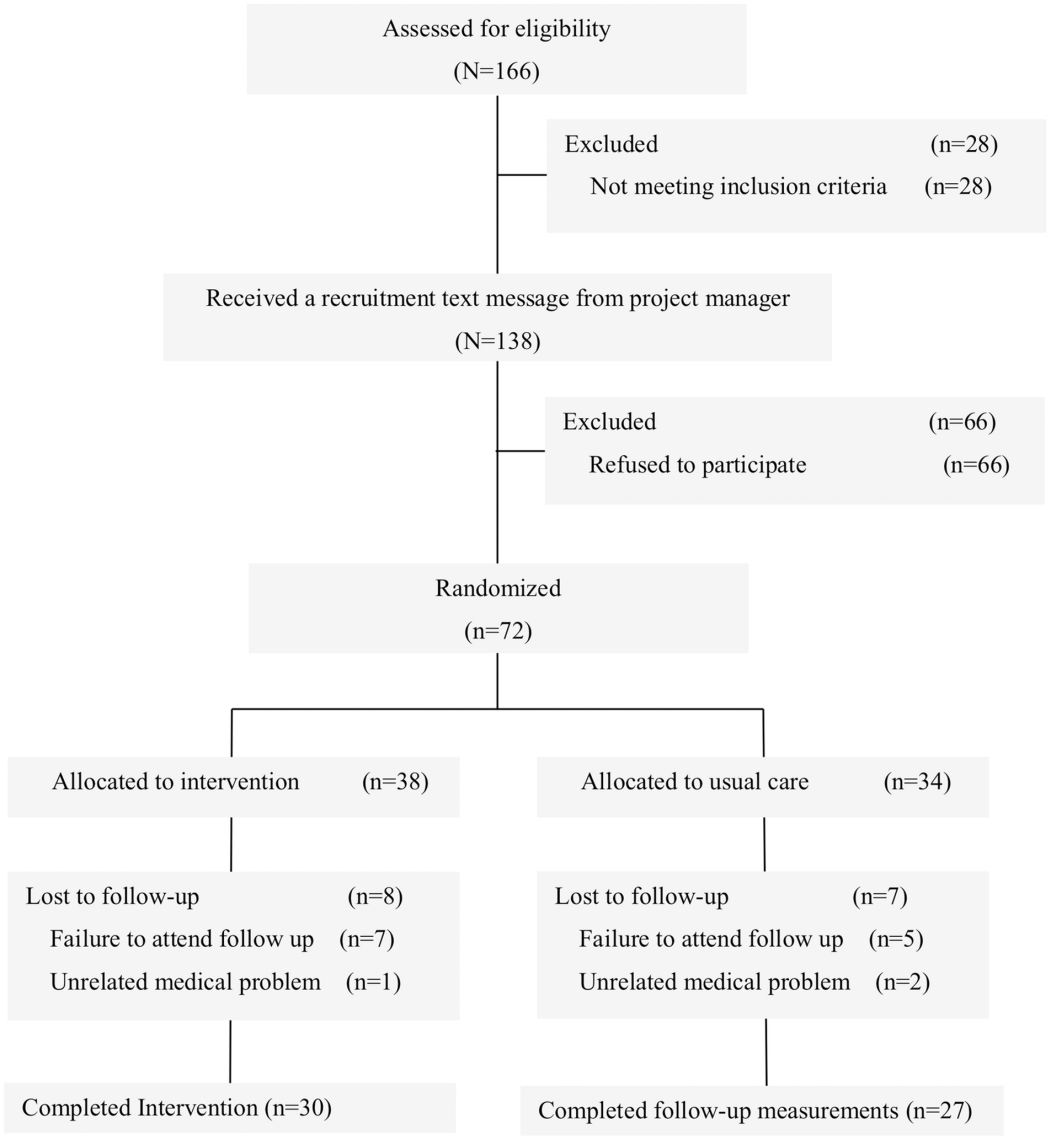
CONSORT diagram.

The flow of participants through the study is provided in Fig 1. In brief, 166 colorectal cancer survivors were assessed for eligibility; 138 (83.1%) were eligible, and 72 (52.2%) were randomly assigned to the exercise (n = 38) or usual care (n = 34) groups. During the intervention period, 8 participants (21.1%) in the exercise group and 7 (20.6%) in the usual care group dropped out. Overall, 57 of 72 participants completed the trial (79.2%). No adverse effects related to the intervention occurred.

### Baseline characteristics

Table 1 shows the baseline demographics and medical profile of participants overall and by group assignment. There were no significant differences in baseline characteristics between groups. The average age was 56.3 ± 9.4 years, 48.6% were male, and 63.9% had been diagnosed with colon cancer. Ninety-three percent of participants had undergone either chemotherapy or both chemotherapy and radiation therapy.

**Table 1:** Baseline demographics and medical profile of participants overall and by group assignment.

Variable	Overall (n = 72)	Exercise (n = 38)	Usual care (n = 34)	*p* value
**Age, y**	56.3 ± 9.4	55.9 ± 8.8	56.8 ± 10.2	0.698
**Male**	35 (48.6)	18 (47.4)	17 (50.0)	1.00
**Weight, kg**	62.9 ± 10.7	63.4 ± 9.0	62.5 ± 12.6	0.725
**Body mass index, kg/m**^**2**^	23.5 ± 3.2	23.6 ± 2.9	23.3 ± 3.6	0.655
**Married**	55 (77.5)	28 (75.7)	27 (79.4)	0.526
**Completed university**	30 (43.5)	19 (52.8)	11 (33.3)	0.458
**Smoking**				0.537
**None**	33 (47.8)	19 (54.3)	14 (41.2)	
**Past**	32 (46.4)	14 (40.0)	18 (52.9)	
**Current**	4 (5.8)	2 (5.7)	2 (5.9)	
**Location of tumor**				0.223
**Colon**	46 (63.9)	27 (71.1)	19 (55.9)	
**Rectal**	26 (36.1)	11 (28.9)	15 (44.1)	
**Tumor stage**				0.103
**II**	33 (45.8)	21 (55.3)	12 (35.3)	
**III**	39 (54.2)	17 (44.7)	22 (64.7)	
**Cancer treatment**				0.397
**Chemotherapy**	58 (80.6)	30 (78.9)	28 (82.4)	
**Chemo+radiotherapy**	9 (12.5)	4 (10.5)	5 (14.7)	
**Time since completed therapy, month**	10.8 ± 7.1	10.9 ± 5.8	10.6 ± 8.4	0.850

Values are presented as mean ± SD or n (%).

### Physical activity level

At baseline, no strenuous-intensity exercise participation was reported in both the exercise and control groups. However, after the 6-week intervention, 20.1 minutes was reported in the exercise group, while only 6 minutes was reported in the control group; the difference was not significant. Moderate-intensity exercise significantly increased from 108.8 ± 196.6 minutes at baseline to 378.2 ± 297.6 minutes after the intervention in the exercise group (vs. usual care group, *p* < 0.001). A total of 26 out of 31 participants (84%) who completed the trial submitted an exercise journal. According to exercise journal analysis, participants in the exercise group walked an average of 11,079 ± 3,540.9 steps per day during the intervention and completed the 30-minute resistance exercises using their own body weight on an average of 5.13 ± 1.85 days per week.

### Physical fitness and body composition

Table 2 presents physical fitness and body composition at baseline and after the 6-week intervention. Participants in the exercise group demonstrated significant improvement in the step test (exercise group, -3.9 vs. usual care group, 2.6; *p* = 0.012) and push-up test (exercise group, 3.0 vs. usual care group, -1.2; *p* = 0.012). No significant difference between groups was observed for changes in the 6-minute walk test (*p* = 0.754), but there was a trend toward a change in chair-to-standing repetitions (exercise group, 0.2 vs. usual care group, -2.2; *p* = 0.137). No significant differences were observed in body weight, BMI, WC, and body fat percentage between groups.

**Table 2:** Changes in physical fitness, body composition, and physical activity level in the exercise and usual care groups (n = 72).

Variable	Exercise (n = 38)		Usual care (n = 34)		Adjusted group differences in mean change		
	Baseline	6 weeks	Baseline	6 weeks	Mean	95% CI	*P*
**Step test**	92.0 ± 13.1	88.0 ± 13.2*	93.0 ± 14.7	95.6 ± 13.6	-7.2	-12.8 to 1.7	0.012
**6-min walk, m/6 min**	576.6 ± 85.5	585.5 ± 82.9	582.5 ± 70.9	594.3 ± 96.7	-3.6	-26.7 to 19.4	0.754
**Chair stand, reps/30 s**	21.1 ± 6.6	21.2 ± 5.2	24.4 ± 6.6	22.2 ± 5.4*	2.0	-0.7 to 4.7	0.137
**Push-ups, reps**	14.2 ± 9.7	17.2 ± 9.6*	16.6 ± 11.8	15.4 ± 11.0	4.3	1.0 to 7.7	0.012
**Weight, kg**	63.4 ± 9.0	63.6 ± 8.9	62.5 ± 12.6	62.9 ± 12.6*	-0.3	-0.8 to 0.1	0.174
**Body mass index, kg/m**^**2**^	23.6 ± 2.9	23.7 ± 3.1	23.3 ± 3.6	23.5 ± 3.6*	-0.15	-0.6 to 0.3	0.534
**Waist circumference, cm**	82.7 ± 8.6	82.6 ± 8.2	81.6 ± 9.8	82.1 ± 10.3	-0.8	-1.9 to 0.4	0.205
**Body fat, %**	27.8 ± 8.5	26.8 ± 8.5	27.5 ± 8.2	27.2 ± 8.4	-1.1	-2.2 to 0.01	0.052
**Strenuous-intensity exercise, min/wk**	0	20.1 ± 78.8	0	6.0 ± 24.2	22.3	-9.9 to 54.6	0.172
**Moderate-intensity exercise, min/wk**	108.8 ± 196.6	378.2 ± 297.6*	128.9 ± 187.0	143.7 ± 174.0	303.7	172.7 to 434.7	<0.001
**Mild-intensity exercise, min/wk**	190.6 ± 225.4	184.6 ± 222.4	197.7 ± 227.8	159.8 ± 187.0	24.9	-83.9 to 133.7	0.649
**Total exercise, min/wk**	296.2 ± 244.1	571.1±382.4*	326.5 ± 294.3	309.4±196.7	348.4	211.4 to 485.4	<0.001
**Total exercise, MET hours/wk**	16.6 ± 14.1	35.6 ± 25.0*	18.5 ± 17.4	18.2±11.6	23.5	14.7 to 32.4	<0.001

Adjusted group difference in mean change was adjusted for baseline value of age, gender, body mass index, education level, location of tumor, tumor stage, and cancer treatment. Values are presented as mean ± SD.

*p < 0.05 difference between baseline and 6-weeks.

## Discussion

The present study examined the feasibility and efficacy of the 6-week home-based exercise program for colorectal cancer survivors. It was hypothesized that the program would significantly increase physical activity level and physical fitness in such patients. As hypothesized, our home-based exercise program significantly increased physical activity levels and significantly improved physical fitness. The fact that the exercise intervention was home based without supervision and that a significant improvement in physical activity level and physical fitness was seen within only 6 weeks is very intriguing.

The goal of the exercise program was to increase physical activity level to 18 MET hours per week over 6 weeks. After 6 weeks, moderate physical activity increased significantly by 269.4 ± 260.6 minutes per week in the exercise group (vs. usual care group, *p* < 0.001). In addition, 73.5% of patients in the exercise group met the exercise goal of ≥18 MET hours per week. The strategy to increase physical activity included an education session that highlighted the importance of physical activity for prevention of cancer recurrence, providence of a daily exercise diary and pedometer, as well as daily text message reminders. It is difficult to distinguish which part of the strategy in particular influenced physical activity behavior, but, collectively, the strategy was successful in promoting physical activity. In a recent study, we identified the importance of oncologists in promoting physical activity among breast and colorectal cancer survivors [19]. In that study, participants who received only oncologists’ exercise recommendations insufficiently increased their physical activity participation, whereas those who received oncologists’ exercise recommendations as well as physical activity education, an exercise journal, and pedometer increased their weekly physical activity participation by an average of 87 minutes. Interestingly, the previous and current studies used very similar home-based exercise interventions, except for the additional daily text message and weekly phone call reminders used in the current study.

Increase in physical activity was accompanied by an improvement in physical fitness and upper body strength. Because our home-based exercise program incorporated a video that included push-up exercise, it is not surprising to observe a significant improvement in upper body strength. The significant increase in number of push-ups performed showed that participants complied with the given exercise program. We also observed significant improvement in physical fitness measured by using the Tecumseh step test. The Tecumseh step test monitors heart rate after 3 minutes of stepping exercise (24 steps per minute) and 1 minute of recovery, and it has been validated to be associated with physical function, visceral fat mass [20, 21], and prevalence of metabolic syndrome [22]. However, we did not observe improvement in the 6-minute walk test, which is more frequently used in cancer survivors [23]. Although both tests can be used to measure physical function and as surrogate measures for cardiopulmonary fitness sometimes, the one important difference between these two tests is the participant’s personal will and endeavor. While the result of the step test (heart rate recovery) is not influenced by personal effort, the result of the 6-minute walk test can be influenced by personal effort. Considering this factor, the result of the step test may reflect physical function much better by independent personal effort or will.

Both physical activity level and physical fitness are closely related to colorectal cancer specific–mortality and all-cause mortality. A recent meta-analysis including 6,348 patients reported that physical activity of any amount reduced colorectal cancer–specific mortality (relative risk [RR], 0.74; 95% confidence interval [CI], 0.58–0.95) and all-cause mortality (RR, 0.65; 95% CI, 0.47–0.92) [8]. Furthermore, there is sufficient evidence showing that cardiopulmonary fitness is associated with prognosis in patients with colorectal cancer. Therefore, increasing physical activity participation and subsequent improvement in physical fitness are important for better prognosis in cancer survivors. Most previous studies have used a supervised exercise intervention when investigating the effect of exercise on cancer-related outcomes. However, supervised exercise interventions can be costly, and many cancer survivors live far from relevant exercise facilities. Therefore, the home-based exercise program used in the current study sheds light on the importance of accessible exercise interventions to increase physical activity level and physical fitness in cancer survivors. Furthermore, we recently reported that 12 week of a home-based exercise program, the same exercise protocol used in the current study, not only increased physical activity and improved physical function, but also significantly reduced circulating insulin levels in colorectal cancer survivors [24]. Knowing that circulating insulin level is associated with prognosis of colorectal cancer [25], exercise induced reduction in circulating insulin level is a beneficial change observed in colorectal cancer survivors.

The current study had several limitations. First, we did not measure maximal oxygen consumption, which is the gold standard for maximal aerobic capacity. Instead, we used the Tecumseh step test and the 6-minute walk test, both of which can be used as surrogate measures for cardiopulmonary fitness. However, caution is required when interpreting the data because these tests better reflect physical function. Second, the sample size was small and since there was no follow-up data, it is not clear about long-term implications and whether these results would be maintained. Therefore, it should be careful in generalizing the result of the study. Thus, further research is required with large sample size and follow-up period. Lastly, although dietary intake is one of important prognostic factors for colorectal cancer survivors, we have not analyzed dietary intake of our participants. However, our main and secondary outcome measures were physical activity and physical fitness, respectively, and therefore, dietary intake would not have influenced our main and secondary outcome variables.

In conclusion, the 6-week home-based mixed aerobic and resistance exercise program significantly improved physical activity level and physical fitness in stage II to III colorectal cancer survivors.

## Supporting information

S1 File Dataset. (SAV)

S2 File CONSORT checklist. (DOC)
